# Phosphatidylcholine-Mediated Aqueous Diffusion of Cellular Cholesterol Down-Regulates the ABCA1 Transporter in Human Skin Fibroblasts

**DOI:** 10.9734/IJBCRR/2015/14058

**Published:** 2015

**Authors:** David Akopian, Ryoko L. Kawashima, Jheem D. Medh

**Affiliations:** 1 Department of Chemistry and Biochemistry, California State University Northridge Northridge CA 91330-8262, USA

**Keywords:** ABCA1, phophatidylcholine, cholesterol transport, cholesterol efflux, aqueous diffusion

## Abstract

ATP-binding cassette protein A1 (ABCA1) is a cholesterol transporter that contributes to the active transport/removal of excess cellular cholesterol. ABCA1 expression is up-regulated when cells accumulate cholesterol.

**Aims:**

The purpose of this study was to determine any correlation between extracellular phospholipid levels and ABCA1 expression and function.

**Methodology:**

Human foreskin fibroblasts were incubated with cholesterol alone or cholesterol and phosphatidylcholine. Total RNA was isolated and subjected to end-point RT-PCR to compare ABCA1 transcript levels. Cell lysates were subjected to Western blot analysis to compare ABCA1 protein levels. Cells were loaded with radiolabeled cholesterol and cellular cholesterol efflux was measured in the presence and absence of apoE, a cholesterol acceptor. ApoE-dependent efflux was calculated as a measure of ABCA1-mediated efflux.

**Results:**

Here we show that incubation of cholesterol-loaded human skin fibroblasts with L-α-phosphatidylcholine (PC) decreases ABCA1 mRNA and protein levels by 93% and 57%, respectively, compared to cells loaded with cholesterol alone. Similarly, PC treatment results in a 25% reduction in ABCG1 mRNA levels compared to cells treated with cholesterol alone, but there is no change in SR-BI transcript levels. Subsequent incubation of phospholipid-treated cells with a cholesterol acceptor such as apoE for 24 hours shows a 65% reduction in ABCA1-mediated cholesterol efflux compared to efflux in cells not treated with PC. During the lipid treatment itself, there is a 2.7-fold greater loss of cholesterol from PC treated cells compared to cells treated with cholesterol alone. Measurement of cholesterol in cellular lipid extracts reveals that cells incubated in the presence of phosphatidylcholine are significantly depleted of cholesterol having only 20% of the cholesterol compared to cells loaded with cholesterol alone.

**Conclusion:**

Thus, phosphatidylcholine facilitates removal of cellular cholesterol, thereby negating the cholesterol-dependent induction of ABCA1 message, protein and function.

## 1. INTRODUCTION

The adenosine triphosphate binding cassette protein A1 (ABCA1) is a cholesterol transporter responsible for the removal of free cholesterol and phospholipids from cells. The resulting flow of cholesterol from peripheral tissues to the liver is termed reverse cholesterol transport (RCT), and influences the plasma lipoprotein profile, particularly HDL levels [1,2]. Mutations in the ABCA1 gene are associated with familial hypoalphalipoproteinemia and a higher incidence of cardiovascular disease. The ABCA1-mediated cholesterol transport is a unidirectional efflux path and requires the presence of extracellular cholesterol acceptors such as lipid-poor apolipoprotein (apo) AI or apo E [2,3]. The lipidation of free apoA-I results in the formation of discoidal pre-beta-migrating HDL particles. The newly acquired cholesterol is esterified by the HDL-associated enzyme lecithin: cholesterol acyltransferase (LCAT) transforming the particle into spherical mature HDL, which carries cholesterol to the liver for degradation [4,5].

Other mechanisms have been described for the transport of cholesterol from cells [3,6]; these include (i) ABCG1-mediated unidirectional efflux to nascent HDL particles, (ii) Bidirectional flux mediated by scavenger receptor class-B type I (SR-BI) and (iii) Aqueous diffusion down a concentration gradient. These three pathways can use HDL as a cholesterol acceptor, whereas ABCA1 does not mediate cholesterol efflux to HDL particles. Likewise, ABCG1 cannot use apoAI or apoE as cholesterol acceptor. Aqueous diffusion and SR-BI-mediated pathways can also efflux cholesterol to phospholipid acceptors. Both processes are bidirectional, with direction of net cholesterol flux determined by the cholesterol concentration gradient. The relative contributions of SR-BI, ABCA1 and ABCG1 pathways in cholesterol efflux are not clear, but depend on the relative expression and activity of the three proteins and the phospholipid and apoprotein content of the acceptor particles.

ABCA1 mRNA and protein levels are up-regulated by cholesterol-loading [3]. Mendez and colleagues observed an increase in apoA-I-mediated energy-dependent cholesterol efflux, distinct from passive diffusion, from cholesterol-enriched human skin firbroblasts [7]. Langmann et al. loaded macrophages with cholesterol by incubation with acetylated LDL and observed an increase in ABCA1 mRNA and protein levels. This effect was erased when cellular cholesterol pools were depleted by incubating the cells with HDL_3_ [8]. Rong and coworkers reported transformation of smooth muscle cells into macrophage-like cells upon loading them with cholesterol, observing a 200% increase in ABCA1 mRNA levels [9].

While ABCA1-mediated efflux is a unidirectional gradient-independent process requiring ATP, passive flux of cholesterol via aqueous diffusion is bidirectional, with the net direction of cholesterol movement dependent on the concentration gradient of cholesterol [10,11]. Many studies have provided evidence for the exchange of cholesterol between synthetic phospholipid vesicles and the plasma membrane of cells. Slotte and colleagues showed that the exchange of cholesterol between phosphatidylcholine vesicles and rat arterial smooth muscle cells occurs by aqueous diffusion and is enhanced in the presence of bovine serum albumin (BSA) [12]. Bartholow and colleagues examined cholesterol efflux from mouse fibroblasts into the medium containing structurally distinct phospholipids, human serum albumin (HSA), or phospholipid-HSA complexes [13]. Their results indicate that phospholipid-albumin complexes are more efficient acceptors of cholesterol than phospholipids and that the degree of unsaturation, charge and length of fatty acyl chains of phospholipids affect the efficiency of efflux.

Like phospholipid liposomes, spherical lipid-rich HDL particles contain phospholipids at the particle-medium interface and are acceptors of cholesterol via aqueous diffusion. LCAT plays an important role in this process by reducing the amount of free cholesterol present in HDL and thus shifting the equilibrium toward further efflux of cellular cholesterol [14,15].

A study by Mendez and colleagues clearly demonstrated the ability of HDL particles to mediate cholesterol efflux via both passive diffusion and an active energy-dependent process [7]. They investigated cholesterol efflux from cultured human skin fibroblasts to a series of acceptors, demonstrating that while apoA-I mediates cholesterol efflux in an energy-dependent manner, phospholipid vesicles and trypsinized HDL, lacking functional apoA-I but having an intact phospholipid monolayer, are acceptors of cholesterol via aqueous diffusion.

The goal of our studies was to investigate the effect of phospholipids on the expression of ABCA1 message and protein, and on ABCA1-mediated cholesterol efflux. In view of the fact that ABCA1 expression is regulated by cellular cholesterol content and that phopsholipids facilitate removal of cellular cholesterol by various mechanisms, we hypothesized that phophatidylcholine may down-regulate ABCA1 expression and function. Our data suggest that phospholipids deplete cellular cholesterol by aqueous diffusion and thereby indirectly down-regulate ABCA1-mediated cholesterol efflux.

## 2. METHODS

### 2.1 Cell Culture

Normal human foreskin fibroblasts (FSF) (GM04390) were obtained from the National Institute of General Medical Sciences Human Genetic Mutant Cell Repository (NIGMS). The cells were maintained in FSF growth medium (DMEM (Cellgro) supplemented with 10% FBS, 2mM glutamine, 50units/mL penicillin, 50μg/mL streptomycin and 10mM HEPES) in a humidified incubator at 37°C and 5% CO_2_. Confluent fibroblasts grown on 12-well plates in FSF growth medium were maintained in DMEM/BSA (FSF growth medium containing 1mg/ml BSA instead of FBS) and used in all treatments.

### 2.2 RNA Isolation and RT-PCR

RNA was isolated using TRI reagent (Sigma) according to the manufacturer’s protocol. Briefly, the cells were washed twice with cold (4°C) PBS, and harvested in TRI reagent by scraping. The total RNA was isolated using chloroform-isopropanol precipitation. The RNA pellet was dissolved in water and the concentration of RNA was determined using a Spectronic Genesys^™^ 5 spectrophotometer. Two μg of RNA per reaction were converted to cDNA using random hexamers, dNTPs (0.44mM) and Moloney Murine Leukemia Virus Reverse Transcriptase (M-MLV RT) (all from Promega). The reagents were mixed and incubated at 25°C for 10 minutes followed by incubation at 42°C for 50 minutes. Second strand synthesis and DNA amplification was accomplished by PCR (25 cycles) using *Taq* polymerase (Promega) using primer pairs shown in Table 1. These indicated amplicon sizes will be obtained only with cDNA and not with any contamination DNA, since the sense and antisense primers are separated by an intron. The PCR reaction mixture contained sense and anti-sense sequence-specific primers (1.9 μM each), dNTPs (380μM), MgCl_2_ (1.9mM), *Taq* polymerase (0.19 u/μL) and 1× *Taq* polymerase buffer. The PCR products were resolved on agarose gel (2%) and quantitation of DNA amplicon was accomplished by ImageJ (NIH) analysis.

### 2.3 Western Blot Analysis of ABCA1 and β-actin

Fibroblasts were chilled, washed twice with cold (4°C) PBS and scraped into the buffer. The cell were pelleted and solubilized in 50 μL of the cell lysis buffer (50 mM Tris-HCl, 2 mM CaCl_2_, 0.1% Triton X-100) containing the serine protease inhibitors, aprotinin (19 μg/mL), benzamidine (1mM) and PMSF (1mM). The amount of protein in the cell lysates was quantitated by the method of Lowry [16].

Equal amounts of protein were resolved under denaturing conditions in a 7.0% polyacrylamide gel and transferred onto polyvinylidine fluoride membrane (Immobilon^™^-P) in transfer buffer (25 mM Tris, 192mM glycine, 15% v/v methanol, pH 8.5) at 30 volts overnight or at 100 volts for 1 hour. The blot was first incubated in blocking buffer (20mM Tris, 150mM NaCl, 2mM CaCl_2_, 1% gelatin, 0.05% TWEEN-20, pH 7.4) overnight at room temperature and then probed with anti-ABCA1 rabbit polyclonal antibody (5 μg/mL, Novus Biologicals) and peroxidase-conjugated goat-anti-rabbit secondary antibody (Jackson Immuno Research Laboratories, Inc.). The Supersignal West Pico chemiluminescent substrate (Pierce) was used to detect the protein bands. The membrane was stripped and reprobed with anti-actin rabbit polyclonal antibody (1.5 μg/mL, Santa Cruz Biotechnology) and goat-anti-rabbit secondary antibody. Results of Western Blot analysis were quantitated using the ImageJ software available at the NIH website.

### 2.4 Lipid Treatments

Cholesterol (99+%) was purchased from Sigma and dissolved in absolute ethanol at 20 mg/mL. L-α-phosphatidylcholine from egg yolk, type XIII-E, 100 mg/mL in ethanol was obtained from Sigma. The lipids were suspended in cell culture media containing 1 mg/mL BSA and sonicated in a bath sonicator for 8 min to form lipid vesicles. For lipid treatments, confluent FSF were washed with PBS and incubated in DMEM/BSA containing 20 μg/mL cholesterol and/or 100 μg/mL PC for 24 hours. Control wells received vehicle (ethanol) alone. The cells were harvested in TRI reagent for isolation of total RNA, or in lysis buffer for protein analysis.

### 2.5 Cholesetrol Efflux Assays

**Fig. 1:**
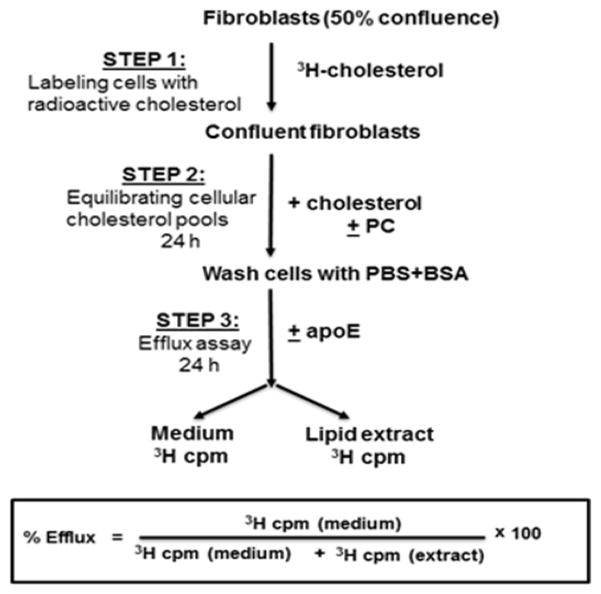
Schematic representation of the cholesterol efflux protocol Step 1: cells were seeded on 12-well plates and labelled with radioactive cholesterol until they reached confluency. Step 2: The labelled cholesterol was equilibrated throughout the cellular cholesterol pools by incubation for 24 hr with unlabelled cholesterol (20 μg/mL). For PC treated cells, 100 mg/mL of L-α-phosphatidylcholine was added during this step. The lipids were suspended in BSA containing medium by sonication to ensure that they dispersed into lipid-protein vesicles. Step 3: ABCA1-mediated cholesterol efflux was activated by incubating in the presence of 12.5 μg/mL of apoE. Control wells without apoE allowed measurement of background ABCA1-independent efflux. The % efflux was calculated using the formula at the bottom

The cholesterol efflux assay was performed as earlier [17]. The experimental protocol is shown schematically in Fig. 1. Fibroblasts were seeded into 12-well cell culture plates and grown to 50% confluence in FSF growth medium prior to addition of either 1 μCi/mL or 0.5 μCi/mL ([1α,2α(n)-^3^H]cholesterol (Amersham Biosciences). The cells were allowed to reach confluence, the radioactive medium was aspirated, the cells were washed twice and incubated with 1 mL of DMEM/BSA containing 20 μg/mL of cholesterol with or without 100 μg/mL PC. After 24 hr, the medium was aspirated and the cells were incubated in DMEM/BSA containing 0.5 mM 8-Br-cAMP with or without 12.5 μg/mL apolipoprotein E for 24 hours. The plates were chilled on ice, the medium was collected into scintillation vials and the cells were washed 4 times with cold (4°C) HEPES-saline BSA (0.03 mM BSA, 138mM NaCl, 5.7 mM KCl, 1.8 mM CaCl_2_, 0.8mM MgSO_4_, 3.7 mM HEPES, pH 7.4) to remove adherent lipids. A 3:2 hexanes: Isopropanol (v/v) solvent was used to extract cellular lipids [18]. Radioactivity was measured using complete counting cocktail (3a70B, Research Products International) and a Beckman, LS 6500 scintillation counter. The amount of the effluxed cholesterol was calculated as: Cholesterol efflux (%) = [radioactivity in the medium/(radioactivity in the medium + radioactivity in the lipid extract)] × 100.

### 2.6 Cholesterol Assay

Total cellular cholesterol was measured using a cholesterol quantitation kit from Abcam (Cambridge MA). The assay uses cholesterol oxidase to generate hydrogen peroxide which reacts with a sensitive cholesterol probe to generate a colored product (λ_max_ = 570 nm). Cellular lipid extract prepared in 500 μL/well of 3:2 hexanes: isopropanol. The extract was dried under nitrogen and the dried lipid pellets were resuspended in 50 μL of the supplied cholesterol assay buffer by vortexing. Samples were treated with cholesterol esterase to hydrolyze any esterified cholesterol to free cholesterol and the resulting total cholesterol was quantified colorimetrically. A standard curve was prepared with cholesterol amounts ranging from 0.25 μg to 2 μg of cholesterol. Results were represented asμg of cholesterol per sample (i.e. per well of cells). Identically plated and treated wells were assayed for total protein content by the method of Lowry to ascertain equal number of cells in each well.

### 2.7 Statistical Analysis

All experiments were repeated at least 3 times. The intensity of DNA and/or protein bands were quantitated using Image J software (NIH). The data were corrected for the housekeeping gene and the percentage change was calculated in treated samples compared to controls. The gel images from one representative experiment are shown with bar graphs (or numeric values) showing averages of 3 or more experiments. For cholesterol efflux experiments (n=3), standard deviation of the sample was calculated. A Student’s t-test, was used to determine statistical significance (P value).

## 3. RESULTS

### 3.1 Phosphatidylcholine Down-Regulates ABCA1 mRNA and Protein Levels

This study aimed to understand the regulation of cholesterol transporters by protein-free phospholipid vesicles. It is already well documented that both ABCA1 and ABCG1 cholesterol transporters are up-regulated by cholesterol loading of cells. SR-BI, which is a bidirectional cholesterol transporter, is not up-regulated by cholesterol. While ABCA1 and ABCG1 are expressed in macrophages and contribute to HDL biogenesis, SR-BI is primarily found on hepatocytes and is involved in the selective uptake of cholesterol esters from HDL particles. We examined the transcription of all three cholesterol transporters after incubation of human foreskin fibroblasts (FSF) with purified Lα-phosphatidylcholine(PC).

**Fig. 2:**
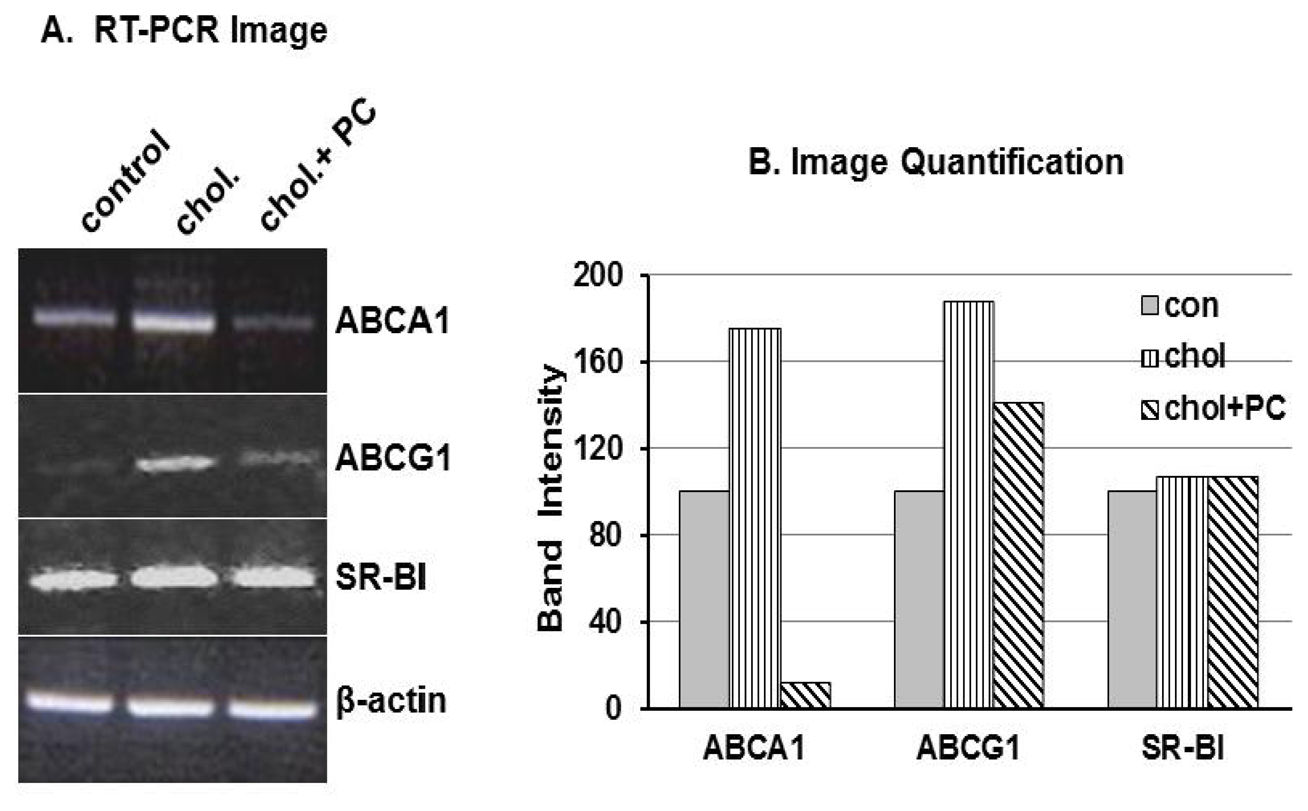
Effect of cholesterol and phosphatidylcholine on the transcription of cholesterol transporters Fully confluent fibroblasts were incubated for 24 hours in DMEM (BSA) in the absence of lipid (control), the presence of cholesterol (20 μg/mL) alone (chol), or a combination of 20 μg/mL cholesterol and 100μg/mL phosphotidylcholine (chol+PC). Total RNA was extracted, and amplicons for ABCA1, ABCG1, SR-BI and β-actin were amplified by RT-PCR (25 cycles), using gene-specific primers (Table 1). (**A**) PCR products were resolved on 2% agarose gels. (**B**) Data in bar graphs were obtained by quantifying the intensity of bands in gel images using Image J. The transporter to β-actin ratios were calculated and values for control cells was set to 100%. The data are representative of at least three experiments with similar results

As seen in Fig. 2, loading the cells with 20 μg/mL of cholesterol resulted in a 75% and 88% increase in the levels of ABCA1 and ABCG1 mRNA levels, respectively. However, when the culture medium was supplemented with 100μg/mL of PC in addition to the cholesterol, the ABCA1 mRNA levels dropped dramatically to only 12% of control levels. Thus, PC negates the induction of ABCA1 by cholesterol. The presence of PC also down-regulated the expression of ABCG1 to 75% of the level in cholesterol only treated cells, but did not reduce it to below the level of control cells. Cholesterol loading or addition of PC did not modulate the level of SR-BI transcript. Incubation of fibroblasts with varying concentration of PC for 24 hours revealed a dose-dependent effect of the lipid on ABCA1 mRNA levels (data not shown) with maximum repression seen at 100 μg/mL.

**Fig. 3:**
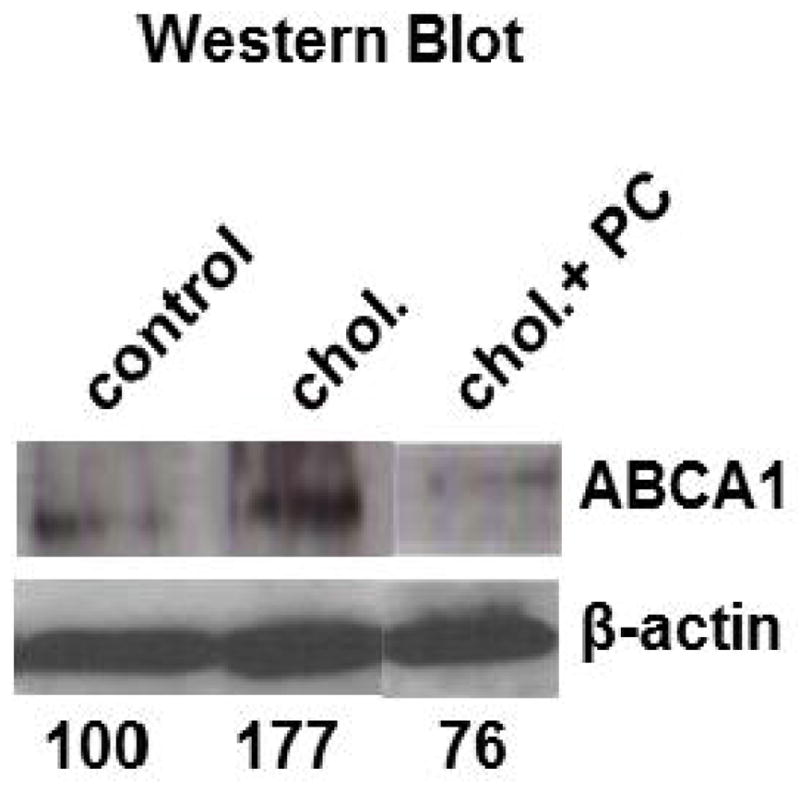
Phosphatidylcholine negates cholesterol-induced ABCA1 up-regulation Cells were treated as described in Fig. 2 and solubilized in lysis buffer. Cellular protein was quantitated and resolved by 7% SDS-PAGE. Western blot analysis of ABCA1 and β-actin was done as described in Methods. The numbers at the bottom represent relative band intensity as quantified by Image J analysis

Phosphatidylcholine down-regulated ABCA1 protein levels as well. Incubation of fibroblasts with cholesterol alone resulted in a 77% increase in ABCA1 protein levels as determined by Western blot analysis (Fig. 3). When fibroblasts were treated simultaneously with cholesterol and PC, the cholesterol-mediated induction of ABCA1 was abolished and ABCA1 protein levels were reduced to 76% of control levels. The intensity of β-actin was used as a control for cellular protein quantification and gel loading.

### 3.2 Phosphatidylcholine Inhibits ABCA1-Mediated Cholesterol Efflux but Facilitates Aqueous Diffusion of Cholesterol

**Fig. 4:**
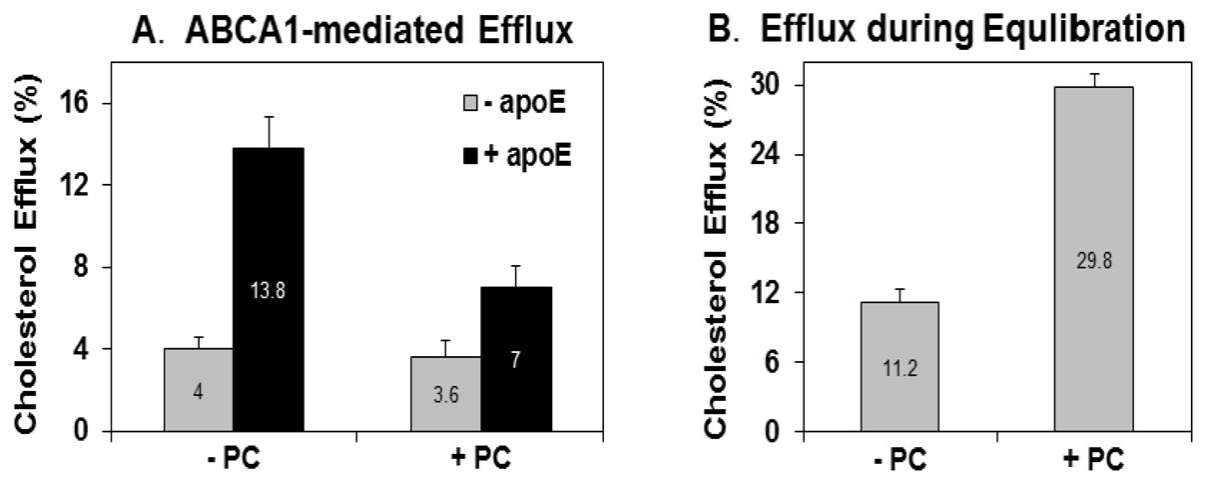
Phosphatidylcholine down-regulates ABCA1-mediated cholesterol efflux but increases the export of cholesterol by aqueous diffusion Cholesterol efflux assay was performed as represented in Fig. 1. FSF were allowed to reach confluence in FSF growth medium containing tritiated cholesterol. The medium was aspirated, the cells were washed once with 1 mL of HEPES/Saline/BSA, and incubated in DMEM/BSA containing unlabeled cholesterol (20μg/mL) (chol) or a mixture of cholesterol (20μg/mL) with phosphatidylcholine (100μg/mL) (**chol** + **PC**) for 24 hours. (**A**) The cells were then washed 4 times with 1 mL of HEPES-Saline BSA. Subsequently, 1 mL DMEM (BSA) with or without apoE (12.5μg/mL) was added to each well. After 24 hr, the medium was collected, the cells were washed, and lipids were extracted. Radioactivity in the medium and the lipid extract was measured using scintillation counting. Percentage of radioactive cholesterol released was calculated from the formula: % Efflux = ^3^H in the medium / (^3^H in the medium + ^3^H in the lipid extract) × 100. Each value is an average of four measurements ± standard deviation. P (13.8, 7) = 0.0012. (**B**) Efflux was measured after step 2 of Fig. 1. Instead of washing away the lipid treatment (equilibration) medium as in A, it was collected and counted for radioactivity. The cells were washed, and lipids were extracted. The percent cholesterol efflux was calculated as in **A**. Each value is an average of three measurements ± standard deviation. P (10.3, 29.8) = 9.1×10^−7^

We then asked if a decrease in ABCA1 mRNA and protein levels translates into reduced ABCA1-mediated cholesterol efflux. The efflux protocol is represented in Fig. 1. Fibroblast monolayers were radiolabeled with [^3^H]-cholesterol until they reached confluence. This was followed by equilibration of labelled cholesterol into various cellular cholesterol pools by incubating cells with cholesterol alone or in the presence of PC for 24 hours. Next, cholesterol efflux was determined by incubating the cells for 24 hours in lipid-free DMEM/BSA in the presence or absence of apoE. ApoE is a required cholesterol acceptor for ABCA1-dependent efflux pathway, hence the difference in efflux between±apoE samples was measured as the fraction mediated by ABCA1. As seen in Fig. 4A, in the absence of PC, the ABCA1 transporter mediated the efflux of 9.8% of cellular cholesterol. In the presence of PC, ABCA1-mediated cholesterol efflux dropped to just 3.4% of total cellular cholesterol. Thus, the presence of PC resulted in a 65% reduction in ABCA1-dependent cholesterol efflux. The ABCA1-independent diffusion of cholesterol, measured in the absence of apoE, remained unaffected by the presence of PC.

### 3.3 Phosphatidylcholine Depletes Cellular Cholesterol

Thus, our results clearly demonstrate that the presence of PC in the cell culture medium reduces ABCA1 mRNA and protein levels and also reduces ABCA1 function. Furthermore, it negates the up-regulation of ABCA1 induced by cholesterol-loading of cells. Based on these observations, we hypothesized that during the equilibration step, PC either depletes cellular cholesterol content, or prevents cholesterol loading of cells. To test this hypothesis, cell culture medium was saved at the end of the equilibration step and cleared of cellular debris. Cellular lipid extracts were also prepared as above after the equilibration step. Both samples were counted for radioactivity and efflux of radiolabeled cholesterol was calculated. This was used as a measure of ABCA1-independent cholesterol efflux. There was a 2.7-fold increase in the amount of the label released by cells in the presence of PC during 24 hours (Fig. 4B). Interestingly, incubation with cholesterol alone did not increase the efflux over control levels (absence of both cholesterol and PC), which was 10.3%+1.5 (data not shown).

**Fig. 5:**
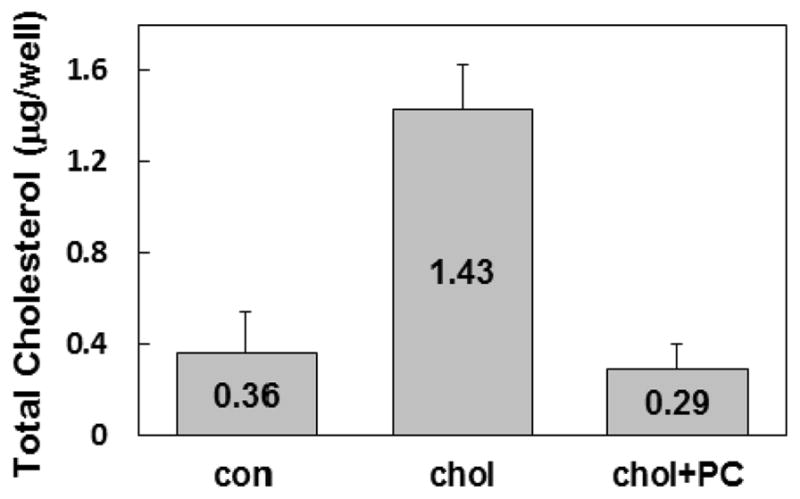
PC depletes cellular pool of cholesterol Total cellular cholesterol content was quantified using a cholesterol quantitation kit from Abcam. Fully confluent fibroblasts were incubated in DMEM/BSA alone (con), or with either 20 μg/mL cholesterol (chol) or a mixture of 20 μg/mL cholesterol and 100 μg/mL PC (chol+PC). The cells were washed 4 times with HEPES-Saline BSA, and lipids were extracted with 3:2 hexanes:isopropanol, dried under nitrogen and resuspended in cholesterol assay buffer. Cholesterol esterase was used to hydrolyze any cholesterol esters in the sample and the resulting total cholesterol was quantified colorimetrically according to the manufacturer’s protocol

To further confirm the finding that incubation of fibroblasts with PC depletes cellular cholesterol, we quantified total cellular cholesterol in lipid extracts of cells after the equilibration step. Cells incubated with cholesterol alone showed a 4-fold increase in total cholesterol compared to control cells incubated without any lipid (Fig. 5). In the presence of both cholesterol and PC, the cellular cholesterol content dropped to 80% of the level in control cells. In similarly treated separate wells, it was ascertained that the lipid treatments did not alter cell viability and cell number and confluency was comparable between treatments. Results are averages of 4 experiments.

## 4. DISCUSSION

In our studies, phospholipids consistently reduced ABCA1 mRNA and protein levels as well as ABCA1-mediated cholesterol efflux from fibroblasts. The most likely mechanism by which phospholipids down-regulate ABCA1-mediated cholesterol efflux is the depletion of cellular pools of cholesterol, nd protein levels. This effect can be explained leading to down-regulation of ABCA1 mRNA a by the cholesterol-accepting capacity of phospholipids. It was previously shown that phospholipid vesicles are efficient acceptors of cellular cholesterol and that the cholesterol-removing capacity of phospholipids increases upon its association with BSA or HSA [13]. In our experiments, both cholesterol and PC were packaged with BSA to form protein-lipid vesicles prior to incubation with fibroblasts.

The mechanism of PC-mediated cellular cholesterol depletion is probably by aqueous diffusion. This conclusion is based on elimination of cholesterol transporter mediated pathways for PC-dependent cholesterol export from cells. The efflux did not require a lipid-poor apolipoprotein, which is an absolute requirement of ABCA1-mediated efflux. ABCG1 mRNA levels were also reduced in the presence of PC, thus, ABCG1 is probably not responsible for the increased PC-mediated efflux. The remaining transporter, SR-BI, employs phospholipids and HDL particles as cholesterol acceptors, thus it could be a good candidate for facilitating cholesterol removal in the presence of PC. However, there was no change in the level of SR-BI transcript, diminishing the likelyhood of this bidirectional transporter being responsible for PC-mediated cholesterol export. Aqueous diffusion is also bidirectional and uses phospholipids as an acceptor for cholesterol, making this the most likely mechanism of PC-mediated cholesterol efflux.

Aqueous diffusion of cholesterol is driven by a concentration gradient of free cholesterol in the cells and in the culture medium. The molar ratio of PC to cholesterol in the medium was 1:4 (100 μg/mL PC and 20μg/mL cholesterol) and there is sufficient BSA in the medium, thus, it is expected that the PC-BSA complexes form multi-lamellar vesicles in association with free cholesterol, shifting the equilibrium toward the removal of cellular free cholesterol. Additionally, the presence of PC in the medium sequesters some of the cholesterol and makes it unavailable for cellular uptake. Since cholesterol-enriching of the cells was shown to up-regulate expression of ABCA1, PC-mediated reduction of cholesterol available in the medium, and depletion of cellular cholesterol are expected to produce the opposite effect. This is consistent with the finding that depletion of the cellular pools of cholesterol by HDL and cyclodextrin was shown to reduce ABCA1-mediated cholesterol efflux [8,10,11]. Our data show that phospholipids are also capable of down-regulating ABCA1 expression by a similar mechanism.

Quantitative analysis of cellular cholesterol levels supports the model of phospholipid-mediated depletion of cellular cholesterol. The cholesterol content of cells was 5-fold lower in cells incubated in the presence of PC compared to those without. These data confirm that a significant amount of cholesterol was removed from the cells in the presence of phospholipids.

Physiologically, phospholipids are most abundant in HDL particles, and HDL particles are potent acceptors of cholesterol via aqueous diffusion and the SR-BI pathways. Our data suggest a role for HDL in regulation of ABCA1-mediated cholesterol efflux *in vivo*. As described earlier, phospholipid- and apolipoprotein-rich HDL particles accept cellular cholesterol via both active lipidation of apoA-I by ABCA1 and passive desorption of cholesterol into the phospholipid monolayer of the particle [2,6]. The results of our studies show that phospholipids of HDL may indirectly deactivate transcription of the ABCA1 gene via depleting cellular cholesterol in the absence of apoA-I. Thus, selective delipidation of plasma, such that neutral lipids are removed, but phospholipids are retained, may result in potent cholesterol acceptors. As suggested by Cham and Chase [19], infusion of such delipidated autologous plasma may be beneficial for the reversal of atherosclerosis.

## 5. CONCLUSION

In conclusion, we showed that phospholipids reduce ABCA1 mRNA and protein levels, decreasing ABCA1-mediated cholesterol efflux. Furthermore, our data suggest that the PC-dependent modulation of ABCA1 occurs via passive efflux of cholesterol into the phospholipid-rich medium and depletion of cellular cholesterol.

## Figures and Tables

**Table 1:** Primer sequences for Reverse-Transcription PCR

Gene and amplicon size (bp)	Primer type	Primer sequence
huABCA1 (157)	Forward	5′-AACAGTTTGTGGCCCTTTTG-3′
	Reverse	5′-AGTTCCAGGCTGGGGTACTT-3′
huABCG1 (317)	Forward	5′-GGTTCTTCGTCAGCTTCGAC-3′
	Reverse	5′-GTTTCCTGGCATTCAGGTGT-3′
huSR-BI (216)	Forward	5′-CTGTGGGTGAGATCATGTGG-3′
	Reverse	5′-GCCAGAAGTCAACCTTGCTC-3′
β-actin (285)	Forward	5′-TCATGAAGTGTGACGTTGACATCCGT-3′
	Reverse	5′-CTTAGAAGCATTTGCGGTGCACGATG-3′
